# A Rare Big Chinese Family With Thrombocytopenia 2: A Case Report and Literature Review

**DOI:** 10.3389/fgene.2020.00340

**Published:** 2020-04-15

**Authors:** Chengning Tan, Limeng Dai, Zhengqiong Chen, Wuchen Yang, Yali Wang, Cheng Zeng, Zheng Xiang, Xiaojie Wang, Xiaomei Zhang, Qian Ran, Hong Guo, Zhongjun Li, Li Chen

**Affiliations:** ^1^Lab of Radiation Biology, Department of Blood Transfusion, The Second Affiliated Hospital, Army Medical University, Chongqing, China; ^2^Department of Medical Genetics, College of Basic Medical Science, Army Medical University, Chongqing, China; ^3^Department of Obstetrics and Gynecology, The Second Affiliated Hospital, Army Medical University, Chongqing, China; ^4^Department of Hematology, The Second Affiliated Hospital, Army Medical University, Chongqing, China

**Keywords:** thrombocytopenia 2, beta thalassemia, hypofibrinogenaemia, *ANKRD26*, *HID1*

## Abstract

Thrombocytopenia 2 (THC2) is one of the most prevalent forms of inherited thrombocytopenia. It is caused by a heterogeneous group of *ANKRD26* gene mutation and shows a heterogeneous clinical and laboratory characteristics. We present a big Chinese family with 10 THC2 patients carrying c.-128G > T heterozygous substitution in the 5-untranslated region of the *ANKRD26* gene. Although the platelets are fewer than 50 × 10^9^/L in 8 THC2 family members, only the proband and her son show a higher WHO bleeding score. The proband and her son are also beta-thalassemia carriers with heterozygous c.52A > T mutation of *HBB*, which might not be associated with the increased bleeding tendency since 3 other family members with low bleeding tendency also carried both *ANKRD26* c.-128G > T and *HBB* c.52A > T mutations. However, the proband and her son also show hypofibrinogenaemia, which is likely the cause of their more severe clinical manifestation. *HID1* c.442G > T mutation was detected not only in these two hypofibrinogenaemia family members but also in the other 8 family members with normal blood fibrinogen levels. Our study suggests that the co-occurrence of other inherited genetic conditions associated with blood coagulation might contribute to the heterogeneity of clinical and laboratory characteristics in THC2 patients. Considering the hematologic and myeloid malignancy predisposition of THC2 patients and a large population of immune thrombocytopenia in China, we urge more attention to be paid to the diagnosis of THC2 patients to avoid misdiagnosis and mistreatment.

## Introduction

THC2 is an autosomal dominant nonsyndromic disorder caused by heterozygous mutation in the 5-untranslated region (5-UTR) of the *ANKRD26* gene (Pippucci et al., 2011). Most of the THC2 patients are characterized by moderate thrombocytopenia, normal hemoglobin level, increased blood leukocyte counts, and mild bleeding tendency (Noris et al., 2014). The platelet size and glycoprotein expression are normal, but the α-granule is deficient in most cases (Noris et al., 2014). Increased TPO level in the serum and dysmegakaryopoiesis in the bone marrow have been observed in all reported cases (Noris et al., 2014). However, the degree of thrombocytopenia and the severity of bleeding diathesis vary among THC2 patients (Noris et al., 2014). The most severe thrombocytopenia patients show a platelet count of 7 × 10^9^/L, while some of the THC2 patients show a normal platelet count of 176 × 10^9^/L (Pippucci et al., 2011). Most of the THC2 subjects show normal blood coagulation, while some show a bleeding tendency between 1 and 4 degrees based on the WHO bleeding score (Pippucci et al., 2011). The potential mechanisms associated with such variable clinical outcomes are yet unknown. We hereby present a big Chinese family with 10 THC2 patients. Among them, 3 subjects who are also beta-thalassemia carriers, 2 other THC2 subjects who carry both beta-thalassemia and hypofibrinogenaemia mutations show a much higher bleeding tendency. Our cases indicate that such variability in clinical manifestation among THC2 patients might be caused by other inherited blood coagulation defect(s) accompanying THC2 in these subjects.

## Case Presentation

**FIGURE 1:**
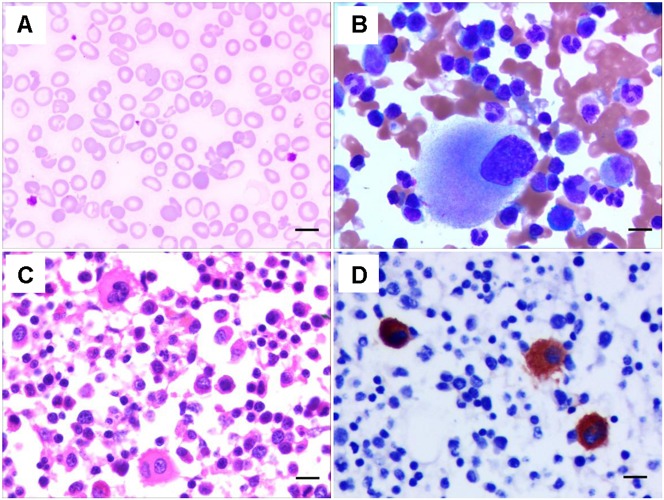
Characteristics of the peripheral blood and bone marrow of the THC2 proband. The peripheral blood smear **(A)** and the bone marrow aspirate smear **(B)** were stained with May–Grünwald-Giemsa. The bone marrow biopsy was stained Hematoxylin and eosin **(C)** and anti-CD61 staining **(D)**. Scale bars represent 10 μm.

A 24-year-old Chinese woman was transferred to our hospital because of increased vaginal bleeding 13 days after cesarean delivery in a local hospital. Complete blood counts upon admission to our hospital showed white blood count (WBC) of 21.84 × 10^9^/L, hemoglobin (HGB) of 48 g/L, and platelet (PLT) of 28 × 10^9^/L. The mean corpuscular volume (MCV) was 67.8. The reticulocyte count was 217.3 × 10^9^/L. The mean platelet volume (MPV) was not detectable. C-reactive protein was 22 mg/L. Thrombin time was 29.1 s, prothrombin time was 15.0 s, activated partial thromboplastin time (APTT) was 38.6 s, fibrinogen (FBG) was 0.48g/L. Thrombelastogram showed a normal *R*-value of 6.1 min, an elongated *K*-value of 8.20 min, a decreased angle value of 30.3 degrees and a decreased MA value of 32.7 mm. Color Doppler ultrasound indicated hematometra. Coomb’s test was positive (+ + ++ for indirect Coomb’s test and 0.5+ for direct Coomb’s test). Her peripheral blood smear showed target cells, teardrop cells as well as schistocytes of red blood cells, and confirmed low platelet count with variable platelet size (Figure 1A). Her bone marrow aspirate smear and biopsy showed increased erythropoiesis, increased leukocytopoiesis and increased megakaryocyte number with hypolobulated nuclei (Figures 1B–D). To correct anemia and improve coagulation function, 6000 mL red blood cell suspension, 4 doses of platelets, 4800 mL fresh freezing plasma, 4800 mL ordinarily frozen plasma, 190 units cryoprecipitation and 13 g human fibrinogen in total were given during her 22-day hospitalization. Blocking therapy was provided by giving intravenous immunoglobulin and dexamethasone. One uterine curettage, two uterine artery embolization, six plasma exchanges, anti-infection therapy, and other symptomatic treatments were also provided. When discharged from the hospital, the patient had a good general condition, minimal vaginal bleeding and healed abdominal incision without bleeding or exudation.

In this case, the patient had a bleeding history in the form of hypermenorrhea, easy epistaxis, and subcutaneous purpura before delivering her son. Her mother and father denied any bleeding history. However, her grandma, one of her aunt and her aunt’s daughter also had frequently subcutaneous purpura. A detailed family history investigation was conducted 3 months later. Blood samples of 29 family numbers were collected with written informed consent obtained from all study subjects or their legal guardians. Hematological parameters indicated that 12 individuals had thrombocytopenia, among them 8 individuals’ platelet counts were fewer than 50 × 10^9^/L. Interestingly, the WHO bleeding scores acquired from them were quite different, only the proband and her son show a higher bleeding tendency (Table 1). Besides, 7 family members are beta-thalassemia carriers with abnormal MCV values, and 2 of them have hypofibrinogenaemia with abnormal FBG values (Table 1).

**TABLE 1:** Clinical and laboratory characteristics of the THC2 family reported in this study.

Subjects	Age	WBC (×10^9^/L)	HGB (g/dL)	MCV (fL)	PLT (×10^9^/L)	MPV (fL)	FBG (g/L)	TPO (pg/mL)	WHO bleeding score	*ANKRD26* Mutation	*HBB* Mutation	*HID1* Mutation
I1	79	6.69	11.6	67.8	117	10.7	3.36	27.14	0	WT	c.52A > T	WT
I2	76	10.25	12.7	102.5	38	9.3	5	225.22	1	c.-128G > T	WT	c.442G > T
I3	72	10.2	13.7	92.1	25	12.5	2.67	NT	1	c.-128G > T	WT	WT
I4	74	5.18	10.6	97.1	110	13.3	3.44	NT	0	WT	WT	WT
I5	69	NT	NT	NT	NT	NT	NT	NT	NT	NT	NT	c.442G > T*
I6	66	3.75	11.2	92	75	14.5	2.71	NT	0	WT	WT	WT
II1	53	8.89	11.7	61.1	37	UD	2.15	133.76	0	c.-128G > T	c.52A > T	c.442G > T
II2	49	7.85	11.1	89.7	104	11.7	NT	110.73	0	WT	WT	WT
II3	50	7.74	9.8	60.8	22	7.9	2.54	150.30	1	c.-128G > T	c.52A > T	c.442G > T
II4	48	7.11	14.1	87.6	122	12.6	2.33	48.91	0	WT	WT	WT
II5	48	3.63	9.7	67.2	157	10	2.74	37.14	0	WT	c.52A > T	WT
II6	45	6.17	14.7	86.2	33	9.9	3.29	210.34	0	c.-128G > T	WT	c.442G > T
II7	38	5.04	12.4	90	151	11.4	2.29	37.95	0	WT	WT	WT
II8	45	7.86	11.7	87.6	19	UD	2.42	NT	1	c.-128G > T	WT	WT
II9	44	6.63	12.6	85.1	213	12.1	2.63	NT	0	WT	WT	WT
II10	41	3.51	11	89.3	131	12.8	2.33	NT	0	WT	WT	WT
II11	40	11.39	14.9	89.3	68	10.5	2.67	NT	0	c.-128G > T	WT	WT
II12	39	3.13	11.3	89.7	111	13.6	2.55	NT	0	WT	WT	c.442G > T
II13	41	4.59	12.9	91.9	83	14.2	2.98	NT	0	WT	WT	WT
II14	41	NT	NT	NT	120	NT	NT	NT	0	WT	WT	WT
II15	39	4.77	10.8	95.2	148	13.6	2.67	NT	0	WT	WT	WT
III1	25	7.24	16.8	96.6	201	9.7	NT	NT	0	WT	WT	WT
III2	24	5.11	6.9	58.9	36	UD	0.89	49.76	4	c.-128G > T	c.52A > T	c.442G > T
III3	24	6.88	10.3	62.5	39	8.3	2.64	85.39	1	c.-128G > T	c.52A > T	c.442G > T
III4	12	6.14	13.4	85.3	147	11.3	2.84	45.01	0	WT	WT	WT
III5	21	6.55	14.3	88.8	145	12.6	2.3	NT	0	WT	WT	WT
III6	17	4.86	14	91.8	200	11.3	2.01	NT	0	WT	WT	WT
III7	12	7.27	12.5	84.1	265	12.4	2.84	NT	0	WT	WT	c.442G > T
III8	18	6.9	12.1	87.4	185	11.7	2.67	NT	0	WT	WT	WT
IV1	0.5	4.98	7.9	69.5	79	UD	1.62	288.579908	2	c.-128G > T	c.52A > T	c.442G > T

Normal values: WBC, white blood cell, 3.5-9.5 × 10^9^/L, HGB: hemoglobin, female 11–15 g/dL, male 12–16 g/dL, MCV, mean corpuscular volume, 82–100 fL PLT, platelets, 125-350 × 10^9^/L, MPV, mean platelet volume, 9–13 fL, FBG, fibrinogen, 2–4 g/L, TPO, thrombopoietin; NT, not tested; UD, undetectable; WT, wild type. *The sample is unavailable from subject I5, we presume he had HID1 c.442G > T mutation since his daughter and grandson had the mutation, while his wife is WT.

**FIGURE 2:**
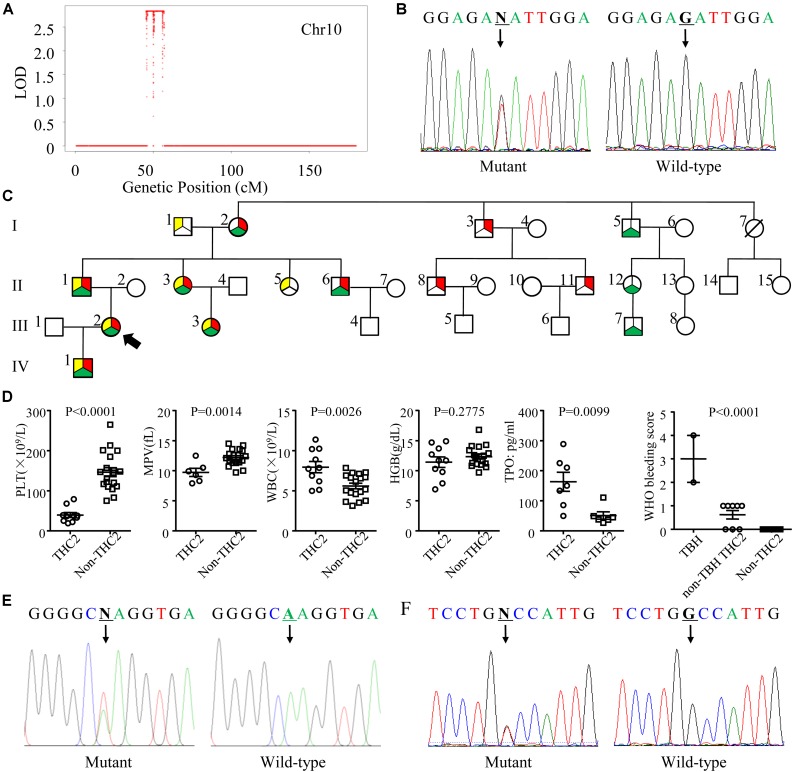
Genetic profile, pedigree, laboratory and clinical characteristics of the THC2 family. **(A)** The linkage analysis including 16 family members was performed with Affymetrix Genome-wide Human SNP 6.0 under a completely penetrant autosomal-dominant model with a disease allele frequency of 0.0003. **(B)** Mutation c.-128G > T in the 5′ UTR of *ANKRD26* was confirmed by Sanger sequencing. **(C)** The pedigree of the THC2 family. Circles, females; squares, males; red in filled symbols indicate individuals with *ANKRD26* (NM_014915) c.-128G > T mutation; yellow in filled symbols indicate individuals with *HBB* (NM_000518.5) c.52A > T mutation; green in filled symbols indicate individuals with *HID1* (NM_030630) c.442G > T mutation. The proband is indicated by an arrowhead; symbol with a diagonal line indicates a deceased individual. **(D)** The platelet (PLT), mean platelet volume (MPV), white blood cell (WBC), hemoglobin (HGB), and thrombopoietin (TPO) levels were compared between THC2 and non-THC2 family members. The WHO bleeding score was compared between TBH (THC2 subjects who carry both beta-thalassemia and hypofibrinogenaemia mutations), non-TBH THC2 (THC2 subjects who do not carry both beta-thalassemia and hypofibrinogenaemia mutations) and non-THC2 (who do not have THC2) family members. **(E)** Mutation c.52A > T in *HBB* was confirmed by Sanger sequencing. **(F)** Mutation c.442G > T in *HID1* was confirmed by Sanger sequencing. Unpaired Student’s *t*-test was used to analyze the differences between 2 groups, one way ANOVA was used to analyze the differences between multiple groups.

To identify the pathogenic mutation(s) of thrombocytopenia, linkage analysis was performed with Affymetrix Genome-wide Human SNP 6.0 under a completely penetrant autosomal-dominant model with a disease allele frequency of 0.0003. Sixteen family members (I1, I2, I3, II1, II2, II3, II4, II5, II6, II7, II8, III2, III3, III4, III5, and III6) were genotyped and the results showed the exceeded genome-wide significance for linkage at 10p12.31-p11.23 with a LOD score of 2.8 (Figure 2A). Whole exome sequencing of 3 family members (I3, II8, and III5) indicated that the pathogenic mutation of thrombocytopenia was c.-128G > T within the 5′ UTR of *ANKRD26* (NM_014915), which has been proven to be the cause of THC2 in a family of Finnish origin (Averina et al., 2017). Sanger sequencing showed that 10 family members among the 12 thrombocytopenia individuals of this parentage carried this mutation of THC2, excepting for family members I6 and II13 (Table 1 and Figures 2B,C). The PLT and MPV level in the THC2 family members were significantly lower than those of non-THC2 family members. The WBC and TPO levels in the THC2 family members were significantly higher than those of non-THC2 family members. No significant difference was observed for the HGB level between THC2 and non-THC2 family members (Table 1 and Figure 2D). We have also demonstrated that the pathogenic mutation of beta-thalassemia was c.52A > T (p.Lys18Ter) of *HBB* (NM_000518.5) (Figure 2E) which is very common in the Chinese population (Yang et al., 2019). And the mutation was detected in all of the 7 family members with abnormal MCV values (Table 1). The likely pathogenic mutation of hypofibrinogenaemia might be c.442G > T (p.Ala148Ser) of *HID1* (NM_030630) (Figure 2F) since some SNPs of *HID1* was reported to be associated with decreased fibrinogen in a GWAS study (Huffman et al., 2015). However, eight family members with *HID1* c.442G > T mutation show a normal fibrinogen level.

## Discussion

THC2 was first reported in 1999, the pathogenic gene was mapped to chromosomal region 10p (Savoia et al., 1999), *MASTL* (Gandhi et al., 2003), and *ACBD5* (Punzo et al., 2010) genes within this region were once been considered to be pathogenic. However, the 5′ UTR mutation of the *ANKRD26* gene was confirmed to be the real cause in 2011 (Noris et al., 2011; Pippucci et al., 2011). A missense mutation c. 473A > G in *ANKRD26* was also reported segregating with thrombocytopenia (Al Daama et al., 2013). However, a mechanistic study showed that it was the failure of *ANKRD26* silencing, rather than the loss of function of ANKRD26 protein during the late stages of megakaryopoiesis caused THC2 (Bluteau et al., 2014). Until now, 75 families involving 267 individuals have been reported as THC2 caused by *ANKRD26* mutation (Table 2). In addition to c. 473A > G, 11 other single nucleotide mutations within the 5′ UTR mutation of *ANKRD26* were confirmed to be pathogenic, they are c.-113, c.-116, c.-118, c.-119, c.-121, c.-125, c.-126, c.-127, c.-128, c.-134, and c.-140 (Table 2). Among them, c.-128 mutation was the most frequently observed one. The G > A substitution was the primary mutation at c.-128, while G > C and G > T mutations were also pathogenic, indicating that the nucleotide G at c.-128 of *ANKRD26* cannot be replaced by any other nucleotides. The same situations were also observed at c.-118C and c.-127A, which suggesting that positions c.-128, c.-118, and c.-127 were critically important for the binding of transcription factors such as RUNX1 and FLI1 (Bluteau et al., 2014; Table 2). There was only one kind of nucleotide exchange reported at c.-113, c.-119, c.-121, c.-125, c.-134, and c.-140, two kinds of nucleotide exchange at c.-116 and c.-126, other kinds of nucleotides exchanges may exist but we cannot determine whether they are pathogenic or not since limited data availability. In this study, we report a big family with 10 THC2 members all with c.-128G > T mutation, further confirming that the G > T at c.-128 of *ANKRD26* is a key pathogenic mutation.

**TABLE 2:** Summary of reported cases of THC2.

Mutation position	Nucleotide exchange	Family (no. of patients)	References
c. 473	A > G	1(10)	Al Daama et al., 2013
c.-113	A > C	1(3)	Noris et al., 2011
c.-116	C > G	1(3)	Noris et al., 2013
	C > T	3(8)	Noris et al., 2011; Pippucci et al., 2011; Perez Botero et al., 2015, 2016
c.-118	C > A	3(8)	Noris et al., 2011, 2013; Boutroux et al., 2015
	C > G	1(1)	Diep et al., 2019
	C > T	7(25)	Noris et al., 2011, 2013; Pippucci et al., 2011; Marquez et al., 2014; Ouchi-Uchiyama et al., 2015; Diep et al., 2019
c.-119	C > A	2(4)	Noris et al., 2011, 2013
c.-121	A > C	1(3)	Noris et al., 2011
c.-125	T > G	3(5)	Noris et al., 2011; Pippucci et al., 2011; Marconi et al., 2017
c.-126	T > C	1(7)	Najm et al., 2013; Ventz et al., 2013
	T > G	3(6)	Noris et al., 2011, 2013; Liu X. et al., 2018
c.-127	A > G	3(13)	Noris et al., 2011, 2013
	A > T	8(30)	Noris et al., 2011, 2013; Pippucci et al., 2011; Boutroux et al., 2015; Vincenot et al., 2016
	A > C	1(6)	Guison et al., 2017
	Del AT	1(6)	Noris et al., 2013; Bluteau et al., 2014
c.-128	G > A	16(69)	Noris et al., 2011, 2013; Pippucci et al., 2011; Ferrari et al., 2016, 2017; Zaninetti et al., 2017
	G > C	2(4)	Noris et al., 2013; Bluteau et al., 2014; Boutroux et al., 2015
	G > T	1(5)	Averina et al., 2017
c.-134	G > A	10(38)	Noris et al., 2011, 2013; Pippucci et al., 2011; Ouchi-Uchiyama et al., 2015; Wang et al., 2018
c.-140	C > G	6(13)	Boutroux et al., 2015; Ferrari et al., 2016, 2017
Total		75(267)	

*Del, deletion.*

**FIGURE 3:**
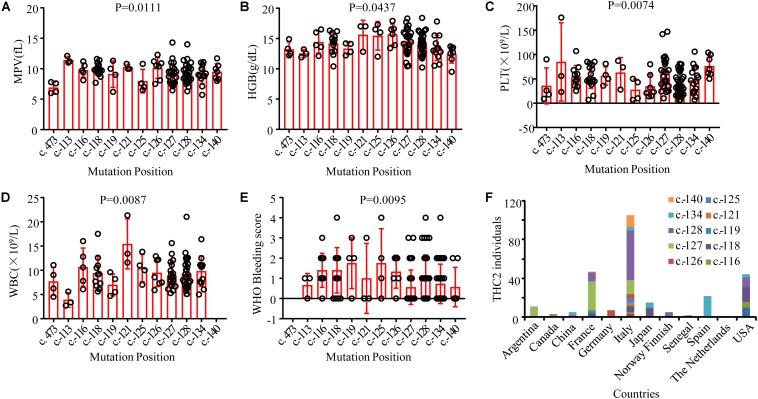
Comparison of laboratory and clinical characteristics among THC2 cases with mutations at different positions in *ANKRD26* and the distribution characteristics of reported THC2 cases in different countries. The mean platelet volume (MPV) **(A)**, hemoglobin (HGB) **(B)**, platelet (PLT) **(C)**, white blood cell (WBC) **(D)** and WHO Bleeding score **(E)** were compared among THC2 cases with different mutation positions in *ANKRD26*. **(F)** The number of THC2 individuals reported in different countries. One way ANOVA was used to analyze the differences between multiple groups.

THC2 in these family members is likely caused by the same gene mutation in *ANKRD26*. Surprisingly, the laboratory and clinical characteristics are highly heterogeneous. To explore the potential underlying mechanisms, we compared the appearance of MPV, HGB, PLT, WBC, and the WHO bleeding scores among these THC2 subjects with 12 *ANKRD26* mutations at different positions (Figures 3A–E). As THC2 is caused by failing to silence *ANKRD26* transcription rather than loss of ANKRD26 protein function (Bluteau et al., 2014), the c. 473A > G mutation in the *ANKRD26* exon region may not be the real pathogenic mutation (Al Daama et al., 2013). It seems more likely that another undefined inherited thrombocytopenia gene mutation might be to blame. For the other 11 *ANKRD26* mutations at other positions, we did not find any obvious association of MPV and HGB levels with any of these mutations (Figures 3A,B). However, we found that subjects with c.-125 or c.-126 mutation show the lowest PLT levels, while those with c.-113 or c.-140 mutation show a relatively higher PLT level (Figure 3C). In contrast, the WBC and WHO bleeding score are relatively low in cases with c.-113 or c.-140 mutation and high in cases with c.-116 to c.-134 mutations (Figures 3D,E). Thus, the region from c.-116 to c.-134 seems to be the core binding site of the *ANKRD26* 5′ UTR with transcription factors RUNX1 and/or FLI1, and the mutation at c.-113 and c.-140 may only partially affect their binding activity (Bluteau et al., 2014).

In addition to the observed laboratory and clinical heterogeneity among the family members with mutations at different positions in *ANKRD26*, we also observed heterogeneity in THC2 family members with mutations at the same position (Figures 3A–E), which could not be explained by the above-described mechanism. In this study, thrombocytopenia was found in 12 family members, 10 of which were confirmed to be THC2 carrying c.-128G > T mutation of *ANKRD26*, the other 2 (the mother I6 and the daughter II13) do not carry this mutation (Table 1), indicating that the thrombocytopenia in the same parentage may be caused by different gene mutations. The real pathogenic mutations in I6 and II13 need further investigation.

Among the 10 THC2 family members, 8 show platelet counts fewer than 50 × 10^9^/L. Interestingly, only the proband and her son show a higher WHO bleeding score (Table 1). The proband and her son are also beta-thalassemia carriers with heterozygous c.52A > T mutation of *HBB*. However, beta-thalassemia patients normally show a hypercoagulation state and manifest thrombotic complications (Seregina et al., 2014). Three other family members who show lower bleeding tendency also carry both *ANKRD26* c.-128G > T and *HBB* c.52A > T mutations. Therefore, the co-occurrence of beta-thalassemia mutation should not be associated with the increased bleeding tendency. Further investigation found that the proband and her son both had hypofibrinogenaemia (Table 1). Fibrinogen was indispensable for blood coagulation, so the co-occurrence of THC2 and hypofibrinogenaemia caused the increased bleeding tendency in the proband and her son. Our findings indicate that the heterogeneity of THC2 clinical manifestations might be influenced by an additional inherited genetic mutation(s) associated with blood coagulation in the subjects who carry the typical THC2 gene mutations.

A GWAS study indicated that some SNPs of the *HID1* gene were associated with decreased fibrinogen (Huffman et al., 2015). In our study, *HID1* c.442G > T mutation was detected in two hypofibrinogenaemia family members and 8 other family members with normal blood fibrinogen levels (Table 1). Why only 20% of the carriers show hypofibrinogenaemia phenotype? Is this phenomenon associated with the co-occurrence of *HID1*, *ANKRD26*, and *HBB* mutations? Further genetic and laboratory investigations will be required to address these questions.

Although the THC2 family members display dysmegakaryopoiesis and micromegakaryocytosis in bone marrow examination (Noris et al., 2011), it was still difficult to distinguish THC2 with other non-inherited thrombocytopenia which has a much higher incidence rate. THC2 patients have been previously reported to be misdiagnosed as having immune thrombocytopenia (ITP) (Noris et al., 2011; Boutroux et al., 2015; Averina et al., 2017) or myelodysplastic syndrome (MDS) (Zaninetti et al., 2017) and receiving potentially damaging immunosuppressive or myelosuppressive treatments, and much worse splenectomy. On the other hand, THC2 cases have been mainly reported in patients from Italy, France and the USA, little is known in China for Chinese patients (Figure 3F). Considering the adult annual incidence rate of ITP is approximately 50–100 new cases per million population per year (Liu X. G. et al., 2018), about 100 thousand individuals may be diagnosed as ITP in China every year. These patients have the risk to be misdiagnosed as THC2 and to be inappropriately treated. In this study, we report a newly identified THC2 family (c.-128G > T) of Chinese origin, in addition to two other reported Chinese THC2 families and 15 other confirmed THC2 patients. Based on the complex clinical manifestation of our reported family members, we believe that THC2 patients in China might be seriously under-diagnosed and/or misdiagnosed. In these scenarios, the patients might either be not treated or mistreated. Further, most of the *ANKRD26* mutations predispose THC2 patients to hematologic and myeloid malignancies (Table 3). We, therefore, urge more attention to be paid to the diagnosis of this rare disease.

**TABLE 3:** Summary of reported malignancy predisposition of THC2 patients.

Mutation position	AL	AML	CML	CLL	MDS	Uterine cancer, eye neoplasia	Visceral malignancies	References
c. 473								
c.-113								
c.-116			+					Noris et al., 2013; Perez Botero et al., 2015, 2016
c.-118	+	+			+			Noris et al., 2011, 2013; Marquez et al., 2014
c.-119								
c.-121								
c.-125	+	+						Noris et al., 2011; Pippucci et al., 2011; Marconi et al., 2017
c.-126								
c.-127	+		+	+				Noris et al., 2011, 2013; Pippucci et al., 2011; Boutroux et al., 2015
c.-128		+	+	+	+	+		Noris et al., 2011, 2013; Pippucci et al., 2011; Bluteau et al., 2014; Ferrari et al., 2016, 2017; Zaninetti et al., 2017
c.-134	+							Noris et al., 2011, 2013; Pippucci et al., 2011
c.-140		+					+	Ferrari et al., 2016, 2017

*AL, acute leukemia; AML, acute myeloid leukemia; CML, chronic myeloid leukemia; MDS, myelodysplastic syndrome; +, malignancies have been reported in THC2 patients.*

## Conclusion

The clinical and laboratory characteristic heterogeneity of THC2 patients could potentially be caused by variations in gene mutation in different family members.

## Data Availability Statement

The raw data supporting the conclusions of this article will be made available by the authors, without undue reservation, to any qualified researcher.

## Ethics Statement

The studies involving human participants were reviewed and approved by the Ethics Committee of The Second Affiliated Hospital of The Army Medical University. Written informed consent to participate in this study was provided by the participants’ legal guardian/next of kin.

## Author Contributions

HG, ZL, and LC designed the study. CT, LD, ZC, WY, YW, CZ, ZX, XW, XZ, and QR performed experiments. LD, HG, and LC performed bioinformatics analyses. LC and LD wrote the manuscript.

## Conflict of Interest

The authors declare that the research was conducted in the absence of any commercial or financial relationships that could be construed as a potential conflict of interest.
